# Efficacy and safety of refined nursing combined with Zhao’s Thunder-Fire Moxibustion in treating patients with gastric cancer of Spleen-Qi Deficiency Syndrome: A clinical study

**DOI:** 10.12669/pjms.40.9.8462

**Published:** 2024-10

**Authors:** Yuelu Yang, Yueling Yang

**Affiliations:** 1 Yuelu Yang, Department of Internal Medicine, Baoding Hospital of Traditional Chinese Medicine, Baoding 071000, Hebei, China; 2 Yueling Yang, Department of Medical Oncology, Baoding No.1 Central Hospital, Baoding 071000, Hebei, China

**Keywords:** Gastric cancer, Quality of life, KPS score, Refined nursing, Spleen-Qi deficiency syndrome, Zhao’s thunder-fire moxibustion

## Abstract

**Objective:**

To investigate the efficacy and safety of refined nursing combined with Zhao’s thunder-fire moxibustion in treating patients with gastric cancer (GC) of spleen-Qi deficiency syndrome.

**Methods:**

This was a retrospective study. Ninety patients with GC of spleen-Qi deficiency syndrome admitted to Baoding Hospital of Traditional Chinese Medicine from February 2022 to March 2023 were randomly divided into the observation group and the control group. Patients in the control group were intervened with XELOX chemotherapeutic regimen + routine nursing. On this basis, patients in the observation group were additionally treated with refined nursing combined with Zhao’s thunder-fire moxibustion. Changes in Traditional Chinese Medicine (TCM) syndrome scores, KPS scores for physical status, and quality of life (QOL) scores were observed and compared between the two groups before and after treatment.

**Results:**

After treatment, the scores of anorexia, fatigue and weakness, postprandial abdominal bloating, loose stools, mental fatigue and laziness to speak decreased significantly in the two groups (*P*< 0.05), and each TCM syndrome score in the observation group was significantly lower than that in the control group (*P*< 0.05). After treatment, KPS and QOL scores in the observation group were significantly higher than those in the control group (*P*< 0.05). The overall nursing satisfaction was 93.3% (42/45) in the observation group, which was significantly higher than 71.1% (32/45) in the control group (*P*< 0.05).

**Conclusion:**

Refined nursing combined with Zhao’s thunder-fire moxibustion can alleviate the clinical symptoms of patients with GC of spleen-Qi deficiency syndrome, reduce chemotherapeutic-induced adverse reactions, with high clinical application value.

## INTRODUCTION

Gastric cancer (GC) is a malignant tumor originating from gastric mucosal epithelial cells. According to statistics, the incidence and mortality of GC in China ranks second and third among malignant tumors. As a key disease entity for cancer prevention and treatment, there are approximately 1.2 million new cases of GC worldwide annually, with China accounting for 40%. In addition, the proportion of early GC is relatively low, and most cases are in disease progression when diagnosed, with an overall five years survival rate of less than 50%.^1^,^2^ Chemotherapy is a major therapeutic option for patients with advanced GC, which can significantly alleviate patients’ clinical symptoms and prolong their survival time, thereby benefiting many GC patients. Nevertheless, chemotherapeutics has a variety of toxic and side effects, such as immunosuppression, neurotoxicity, bone marrow suppression and gastrointestinal reactions, which seriously reduce the quality of life (QOL) of patients, usually leading to their despair of treatment and extremely low desire for survival.^3^-^5^ Consequently, it highlights the importance of carrying out research to effectively improve the QOL of patients with advanced GC. Refined nursing originated from the refined management concept in European and American countries at the beginning of the 20th century. It was proposed by Frederick Winslow Taylor, the father of scientific management, who advocated for scientific, standardized and refined management to reduce management and communication costs, as well as to minimize resource consumption.

At present, refined nursing in China is often studied as a single treatment. By integrating moxibustion, acupuncture and traditional Chinese medicine (TCM), Zhao’s thunder-fire moxibustion is an innovative open-fire over skin moxibustion therapy developed through reforming moxibustion medicine formula and usage based on Zhao’s thunder-fire miraculous needle. Its main working principle is to utilize the pharmaceutical chemical factors, heat and infrared radiation force produced by the burning of drug powder, jointly to achieve the effects of warming meridians and unblocking collaterals, supporting healthy energy, activating blood circulation and removing blood stasis, and eliminating goiters and tumors through the propagated sensation along meridians of acupoints and collaterals.^6^ Moreover, there are also few clinical studies on the application of refined nursing combined with Zhao’s thunder-fire moxibustion both domestically and internationally. Therefore, for the first time, the present study investigated the efficacy and safety of refined nursing combined with Zhao’s thunder-fire moxibustion in treating patients with gastric cancer (GC) of spleen-Qi deficiency syndrome, in the expectation of promoting the clinical research and application of the proposed combined therapy in China.

## METHODS

This was a retrospective study. The subjects of this study were 90 patients with GC of spleen-Qi deficiency syndrome who were admitted to Baoding Hospital of Traditional Chinese Medicine from February 2022 to March 2023. Included patients were further divided into the observation group and the control group using a completely random number table, with 45 cases in each group. The observation group included 25 males and 20 females, with an age of 50-73 (65.8 ± 4.3) years and a course of disease of 1-6 (2.4 ± 0.7) years. The tumors were located at the gastric antrum (n = 17), gastric fundus (n = 19) and gastric body (n = 9). Twenty-six patients were in TNM stage III and 19 in TNM stage IV. In the control group, there were 27 males and 18 females, aged 52-74 (65.5 ± 4.1) years, with a course of disease of 1-7 (2.6 ± 0.8) years. The tumor locations were the gastric antrum (n = 18), gastric fundus (n = 17) and gastric body (n = 10). The TNM stage was classified into Stage-III (n = 28) and stage IV (n = 17). No statistically significant differences were found in the above data between the two groups (*P* > 0.05), suggesting high compatibility. The study was approved by the Institutional Ethics Committee of Baoding Hospital of Traditional Chinese Medicine (No.:2022006; date: October 12, 2022), and written informed consent was obtained from all participants.

### Diagnostic criteria:

The Western medicine diagnosis of GC conformed to the stipulation of the: *Diagnosis and Treatment Standards for Common Malignant Tumors in China-Volume 4-Gastric Cancer*^7^, and was confirmed by clinical, pathological and imaging examinations. The TCM diagnosis referred to the stipulation of spleen-Qi deficiency syndrome in the *Guiding Principles for Clinical Research on New Drugs of Traditional Chinese Medicine*.^8^ Among them, the primary symptoms were fatigue and weakness, anorexia, postprandial abdominal bloating and loose stools, and the secondary symptoms included mental fatigue, laziness to speak, abdominal pain, tastelessness, nausea, vomiting, puffiness of the limbs and sallow complexion, accompanied by pale tongue, enlarged tongue, tooth-marked tongue, thin and white tongue coating, as well as thready and weak pulses.

### Inclusion criteria:

- Patients who met the Western medicine diagnostic criteria of GC and the TCM criteria for disease and syndrome differentiation;
- Age of 20-75 years;
- GC TNM stage III-IV;
- Karnofsky Performance Scale (KPS) score > 60;
- Expected survival time > 6 months;
- Complying with the principle of informed consent.

### Exclusion criteria:

- Patients diagnosed with other intragastric tumors such as gastric stromal tumor and gastric lymphoma;
- Patients complicated with primary malignant tumors at other sites;
- Severe organic diseases of the heart, liver and kidney;
- Mental and blood diseases, allergy to treatment in this study;
- With poor therapeutic compliance and failure in treatment.

Patients in the control group were treated with XELOX chemotherapeutic regimen after admission. The specific regimen was as follows: intravenous drip of oxaliplatin injection (Jiangsu Hengrui Medicine Co., Ltd., NMPN: H20050962, specification: 100 ml: 0.1 g) at 130 mg/m^2^ on the 1st d, and oral administration of Capecitabine Tablets (Shanghai Roche Pharmaceuticals, NMPN: H20073024, specification: 0.5 g) on the 1st-14th d (1.0 mg/m^2^, two times/d). A total of two courses of treatment were conducted, with three weeks as one course and a time interval of one week. In addition, routine nursing was given as follows:

- ***Dietary guidance:*** multiple meal with small amount for each dietary plan to ensure the normal digestive function of these patients;
- Regular disinfection and protection, and guiding patients to eng age in moderate physical exercise to promote immune enhancement;
- Close monitoring of patients’ vital signs, and immediate treatment in case of chemotherapeutic responses.
- Based on the chemotherapy used in the control group, patients in the observation group were additionally treated with refined nursing combined with Zhao’s thunder-fire moxibustion. Refined nursing measures were introduced as follows:
- Establishment of a specialized nursing team including an attending physician, a head nurse and experienced nurses, who were qualified after standardized training and passing the examination;
- ***Development of a nursing plan:*** corresponding nur
- ***Health education:*** with the development of a clinical pathway for health education, the content of education was refined from four aspects of admission, hospitalization, discharge and follow-up, the family members were encouraged to participate in the education.

Psychological nursing: patients’ awareness of this disease was evaluated to identify the presence of psychological problems such as fear and anxiety, and personalized psychological nursing plans were developed to timely address patients’ psychological problems, so as to maximize patient satisfaction in nursing work. Zhao’s thunder-fire moxibustion was performed as follows: patients were informed to keep the supine position, with bilateral Zusanli, Shenque, Zhongwan, Xiawan and Tianshu acupoints fully exposed. Then, the moxa for Zhao’s thunder-fire moxibustion (moxa stick specification: 2.8 cm × 10.0 cm) was ignited and placed in the corresponding tool for moxibustion. Using the reinforcing method, moxibustion was applied on the above acupoints from top to bottom, 3-7cm away from the skin, for 5-10 minutes. After the patients adapted to the temperature of the thunder-fire moxibustion, its distance to the skin was narrowed based on the patients’ skin tolerance. It was advisable to apply the moxibustion to the skin with slight redness. From the 1^st^ day of chemotherapy, moxibustion was applied continuously for two chemotherapy cycles, once a day for 20 min each time.

### Observation indicators: TCM syndrome score:

According to previously reported criteria^8^, the symptoms of GC patients with spleen-Qi deficiency syndrome were scored. The symptoms were scored as zero point (no), one point (mild), two points (moderate) and three points (severe). Patients with higher total scores might have severer spleen-Qi deficiency syndrome.

### KPS score:

The patients’ physical status before and after treatment was assessed using the KPS, with a score of 0-100 points. Patients with higher scores would have a better physical condition.

### QOL score:

Using the QOL scoring scale, the QOL of the enrolled patients was assessed before and after treatment. Patients with higher scores might have better QOL. The total score was 60 points, with 51-60 points as excellent, 41-50 as good, 31-40 as moderate, 21-30 as poor, and ≤ 20 as extremely poor.

### Nursing satisfaction:

Patients’ nursing satisfaction was assessed using the self-designed satisfaction questionnaire by our hospital, which was divided into three parts: very satisfied, basically satisfied, and dissatisfied. Overall satisfaction = very satisfied + basically satisfied.

### Short-term efficacy:

Referring to the Response Evaluation Criteria in Solid Tumors (RECIST)^9^, the short-term efficacy was evaluated as complete remission: the target lesion basically disappeared; partial remission: the baseline sum of the longest diameters (SLD) of the target lesion decreased by ≥ 30%; disease stabilization: the baseline SLD of the target lesion decreased by < 30% or increased by < 20%; and disease progression: the baseline SLD of the target lesion increased by ≥ 20%. Overall clinical benefit = complete remission + partial remission + disease stabilization.

Based on the evaluation criteria for toxic and side effects proposed in the NCI-CTCAE 4.0.3^10^, this study counted the incidences of grade III and IV adverse events such as nausea and vomiting, leukopenia, hepatic and renal dysfunction, peripheral neurotoxicity and hemoglobin reduction. The cut-off point for data analysis was on March 2023, during the three-month follow-up of this study, the survival rate was 100%.

### Statistical Analysis:

All data in this study were processed using SPSS22.0 software. The enumeration data were analyzed by the *χ^2^* test, and the ranked data by the correlated-sample rank-sum test. The quantitative data conforming to the normal distribution were expressed as mean ± standard deviation (*χ̅*±*S*), and analyzed using the *t*-test with homogeneity of variance and the corrected *t*-test with unequal variances. *P*< 0.05 was considered statistically significant.

## RESULTS

After treatment, the scores of anorexia, fatigue and weakness, postprandial abdominal bloating, loose stools, mental fatigue and laziness to speak decreased significantly in the two groups (*P*< 0.05), and each TCM syndrome score in the observation group was significantly lower than that in the control group (*P*< 0.05), as shown in Table-I.

**Table-I:** Comparison of TCM syndrome score between the two groups before and after treatment (*χ̅*±*S*).

Groups	N	Anorexia		Fatigue and weakness		Postprandial abdominal bloating	

		Before treatment	Six weeks after treatment	Before treatment	Six weeks after treatment	Before treatment	Six weeks after treatment
Observation group	45	2.06±0.52	1.41±0.31^① ②^	2.11±0.56	1.42±0.33^① ②^	2.09±0.53	1.38±0.25^① ②^
Control group	45	2.08±0.53	1.70±0.35^①^	2.13±0.58	1.72±0.35^①^	2.08±0.58	1.65±0.30^①^

*Loose stools*				*Mental fatigue and laziness to speak*			

*Before treatment*	*Six weeks after treatment*		*Before treatment*			*Six weeks after treatment*	

2.03±0.33	1.35±0.19^① ②^		2.15±0.29			1.33±0.18^① ②^	
2.05±0.31	1.69±0.22^①^		2.17±0.28			1.65±0.21^①^	

***Note:***

① Compared with before treatment, *P* < 0.05;

② Compared with the control group, *P* < 0.05.

After treatment, KPS and QOL scores increased significantly in the two groups (*P* < 0.05), and KPS and QOL scores in the observation group were significantly higher compared with those in the control group (*P*< 0.05, Table-II). After treatment, the overall nursing satisfaction of the observation group was significantly higher than that of the control group (*P* < 0.05), as presented in Table-III.

**Table-II:** Comparison of KPS and QOL scores between the two groups before and after treatment (*χ̅*±*S*).

Groups	N	KPS score		QOL score	

		Before treatment	Six weeks after treatment	Before treatment	Six weeks after treatment
Observation group	45	64.55±5.26	79.51±6.61^① ②^	26.11±4.03	40.03±4.15^① ②^
Control group	45	63.78±5.30	69.11±6.18^①^	26.13±4.05	34.27±4.09^①^

***Note:***

① Compared with before treatment, *P* < 0.05;

② Compared with the control group, *P* < 0.05.

**Table-III:** Comparison of nursing satisfaction between the two groups after treatment [N (%)].

Groups	N	Very satisfied	Basically satisfied	Dissatisfied	Overall satisfaction
Observation group	45	26(57.8)	16(35.5)	3(6.7)	42(93.3)^①^
Control group	45	10(22.2)	22(48.9)	13(28.9)	32(71.1)

***Note:***

① Compared with the control group, X^2^ = 7.601, P < 0.05.

The overall clinical benefit rate of the observation group was significantly higher than that of the control group (*P*< 0.05, Table-IV). The overall incidence of grade III and IV adverse events such as nausea and vomiting, leukopenia, hepatic and renal dysfunction, peripheral neurotoxicity and hemoglobin reduction in the observation group was significantly lower than that in the control group (*P <* 0.05, Table-V).

**Table-IV:** Comparison of clinical efficacy between the two groups 6 weeks after treatment [N (%)].

Groups	N	Complete remission	Partial remission	Disease stabilization	Disease progression	Overall clinical benefit
Observation group	45	0	26(57.8)	8(17.8)	11(24.4)	34(75.6) ^①^
Control group	45	0	16(35.6)	9(20.0)	20(44.4)	25(55.6)

***Note:***

① Compared with the control group, X^2^ = 3.986, P < 0.05.

**Table-V:** Comparison on the incidence of grade III and IV adverse events during treatment between the two groups [N (%)].

Groups	N	Nausea and vomiting	Leukopenia	Hepatic and renal dysfunction	Peripheral neurotoxicity	Hemoglobin reduction	Total
Observation group	45	2(4.4)	1(2.2)	1(2.2)	1(2.2)	1(2.2)	6(13.2)^①^
Control group	45	4(8.9)	4(8.9)	4(8.9)	3(6.9)	4(8.9)	19(42.5)

***Note:***

① Compared with the control group, X^2^ = 9.360, *P* < 0.05.

## DISCUSSION

The study has confirmed that the mechanism of moxibustion at Zusanli in treating digestive system diseases may lie in that the local receptors of the acupoint can conduct relevant nerve action potentials to the central nuclei through meridians of the nerve-endocrine system, stimulating related substances or molecules, and eventually playing a role in improving gastrointestinal function.^11^ Directly or indirectly connecting to the viscera, the eight extraordinary meridians and the twelve meridians, the Shenque acupoint is the general hub of the whole-body meridians, which is capable of regulating the functions of the meridians and viscera throughout the body. Here, there is no subcutaneous adipose tissue, and the peritoneum is directly connected to the fascia of the skin. Moreover, this acupoint is surrounded by many nerves and blood vessels. Drug or physical stimulation of Shenque can effectively stimulate nerve endings, and then regulate the physiological activities of the viscera through the conduction of the nervous system, thereby enhancing the immune function of the body and the ability to resist diseases.^12^ Furthermore, Zhongwan is the front-mu point of the stomach and the influential point of fu-organs, where the Ren channel intersects with the stomach meridian.

Treatment of epigastric diseases based on this acupoint plays the therapeutic action of the nearby acupoint, with the functions of strengthening the spleen and harmonizing the stomach, and supplementing Qi and unblocking meridians.^13^ Xiawan belongs to the Ren channel and is mainly applied for treating gastrointestinal diseases. Tianshu belongs to the stomach meridian of foot-Yangming, and is the front-mu point of the large intestine, with the functions of promoting blood circulation and dispersing stasis, regulating the flow of Qi to alleviate pain, and clearing dampness and heat. Collectively, the combination of the above acupoints can support healthy energy, strengthen the spleen and harmonize the stomach, and promote blood and Qi circulation.

GC belongs to the category of “accumulation” and “epigastric pain” in TCM.^14^,^15^ Indeed, chemotherapy can prolong the survival of GC patients, which, however, may have a great destructive effect on the immunity and resistance of these patients, often leading to deficiency of vital-Qi, poor QOL and low desire for survival after chemotherapy. Therefore, it is particularly important to develop alternatives that can improve the QOL of patients with advanced GC.^16^ Refined nursing originated from the refined management concept in European and American countries, which can minimize resource consumption and management costs. In this way, it is beneficial for enhancing the nursing quality of hospitals, improving patients’ medical experience, reducing complications, and promoting prognosis.^17^,^18^ Zhao’s thunder-fire moxibustion is a traditional open-fire over skin moxibustion therapy integrating acupuncture, moxibustion and medicine, which was listed as a key new technique promotion project by the State Administration of Traditional Chinese Medicine in 2010. It is easy to operate, needs no metabolism by the human body and does not cause discomfort, and the delightful scent generated during burning can enhance the comfort experience of patients. Thunder-fire moxibustion can reach a local temperature of 240°C, with strong efficacy and extremely high permeability, which can promote the rapid formation of high-concentrating permeation zones of pharmacochemical molecules on the skin surface, greatly improving the therapeutic effect of hot moxibustion,^19^,^20^ which is commonly used to treat digestive system diseases.

### Limitations:

It includes a small number of patients who were included and no long-term follow-up was conducted. In view of this, more patients should be included and follow-up time should be increased in future studies to further validate the effects of Zhao’s thunder-fire moxibustion and refined nursing.

## CONCLUSION

In conclusion, refined nursing combined with Zhao’s thunder-fire moxibustion can improve the QOL of patients with GC of spleen-Qi deficiency syndrome and reduce chemotherapeutic-induced adverse reactions, with important clinical application value.

### Authors’ Contributions:

**YY** carried out the studies, data collection, performed the statistical analysis, participated in its design.

**YY** drafted the manuscript, participated in acquisition, analysis, or interpretation of data and draft the manuscript.

All authors read and approved the final manuscript, and are responsible and accountable for the accuracy or integrity of the work.
